# Non-Contact Measurement of Blade Vibration in an Axial Compressor

**DOI:** 10.3390/s20010068

**Published:** 2019-12-21

**Authors:** Radoslaw Przysowa, Peter Russhard

**Affiliations:** 1 Instytut Techniczny Wojsk Lotniczych (ITWL), ul. Księcia Bolesława 6, 01-494 Warszawa, Poland; 2 EMTD Ltd., 22 Woods Meadow, Derby DE72 3UX, UK; peter.russhard@emtd-measurement.com

**Keywords:** blade vibration, blade tip-timing, rotating stall, axial compressor, blade health monitoring, least squares, bladed disc dynamics

## Abstract

Complex blade responses such as a rotating stall or simultaneous resonances are common in modern engines and their observation can be a challenge even for state-of-the-art tip-timing systems and trained operators. This paper analyses forced vibrations of axial compressor blades, measured during the bench tests of the SO-3 turbojet. In relation to earlier studies conducted in Poland with a small number of sensors, a multichannel tip-timing system let us observe simultaneous responses or higher-order modes. To find possible symptoms of a failure, blade responses in a healthy and unhealthy engine configuration with an inlet blocker were studied. The used analysis methods covered all-blade spectrum and the circumferential fitting of blade deflections to the harmonic oscillator model. The Pearson coefficient of correlation between the measured and predicted tip deflection is calculated to evaluate fitting results. It helps to avoid common operator mistakes and misinterpreting the results. The proposed modal solver can track the vibration frequency and adjust the engine order on the fly. That way, synchronous and asynchronous vibrations are observed and analysed together with an extended variant of least squares. This approach saves a lot of work related to configuring the conventional tip-timing solver.

## 1. Introduction

Non-contact blade vibration measurement, known as blade tip-timing (BTT) or Non-Intrusive Stress Measurement System (NSMS), is a technique for determining dynamic blade stresses to ensure the structural integrity of bladed disks in jet engines and stationary turbines [1,2]. It can be used in axial or radial compressors [3] and unshrouded or shrouded turbines [4]. The method uses several sensors mounted circumferentially to precisely determine temporary positions of each blade tip in every rotation [5]. Model-based data processing is necessary to determine the real amplitude and vibration [6,7]. BTT solutions used by industry are still based on algorithms established at the end of the 20th century. Most vibration surveys rely upon traversing each of the resonances. Analysing responses at a constant speed is more difficult to achieve.

In most applications, undersampled tip deflection signals are generally processed by the two groups of methods: (1) Fourier spectral analysis, e.g., all-blade spectrum, (2) least squares fitting (LSF). Fourier transform methods compromise time and frequency resolution and involve significant post-processing to reconstruct the real spectrum. They are now used primarily to identify the presence of non-integral vibration and to determine nominal frequency and nodal diameter. This data seeds least squares fitting [8]. LSF is now the main method used in most of BTT systems to extract the real amplitude and phase for successive revolutions. It can be used either circumferentially to fit the blade mode or axially to fit the mode shape - each has a different strategy for conversion to stress [9]. The models for this can be simplistic sine fitting [10] or more complex ones derived from the finite element method (FEM) during the calibration and validation process used with BTT.

Alternative methods introduced in the previous decade such as auto-regressive [11], spectral estimation using nonuniform sampling [12], or full-signal analysis using many points per blade pass [13], were too complex or not efficient enough to leave the labs and be widely used by the community. These alternative methods provide little additional capability to the BTT technology but are often revisited by researchers [14,15,16] in the hope of finding methods for in-service blade health monitoring (BHM), where the blade sets are already well understood.

A significant number of papers, introducing new BTT models and algorithms such as a new two-parameter plot method [17], convolutional neural networks [18], aliasing reduction [19,20], sparse representation, and compressed sensing [21], were published recently, but they can be applied in real life to a limited extent. Several newly introduced algorithms work well only with simulated or rig acquired data, usually with a single response of the first mode. Further efforts are needed to make them more mature to better deal with noise and weak or more complex responses.

Validation against simulation is not accepted for certification of engines [22]. BTT technique was already used in component certification tests, where the evidence to calculate a meaningful value of stress was produced [23]. However, a documented validation of the measurement system and ability to estimate uncertainty are still missing in many BTT applications.

This paper presents robust and efficient methodology of processing tip-timing data which produces vibration results of known and controlled uncertainty. It is demonstrated on two datasets acquired in a realistic test-cell environment.

First, stack pattern is calculated to check alignment, validate measurement data, and avoid misinterpreting it, especially when strong low-order excitation force is present. Then, an extended method of least squares is used to analyse coinciding integral and non-integral blade vibrations together. The Pearson coefficient of correlation between the measured and predicted tip deflection is calculated to evaluate fitting results.

Finally, to study interaction between the rotating stall and integral resonance, a new modal solver is used, which can track the modal frequency and adjust the engine order on the fly, producing results similar to strain gauges. Measurement uncertainty is calculated for any point on the tracked order response to assess the overall results.

## 2. Materials and Methods

**Figure 1:**
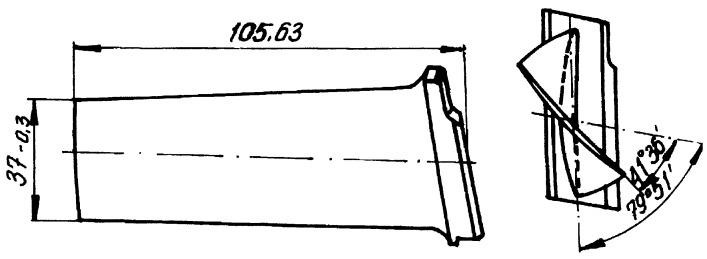
Geometry of the first-stage blade.

This work analyses forced vibrations of axial compressor blades, measured with tip timing system during the bench tests of the SO-3 turbojet. SO-3 is a first generation single-spool engine with the seven-stage axial compressor and no variable stator vanes. It rotates anticlockwise (looking from aft forward) with the maximum speed 15,600 rpm. There are 28 blades in the first stage with dovetail roots (Figure 1), made of 18H2N4MA steel and painted yellow for protection against corrosion. The stage is subsonic, preceded by three struts and 44 constant inlet guide vanes (IGV), and followed by 34 downstream vanes.

The conducted engine tests were aimed to characterise blade vibrations i.e., identify existing responses, especially high-order ones. This is often necessary when investigating blade failure in legacy compressors or turbines, when design data are not available or not reliable.

**Figure 2:**
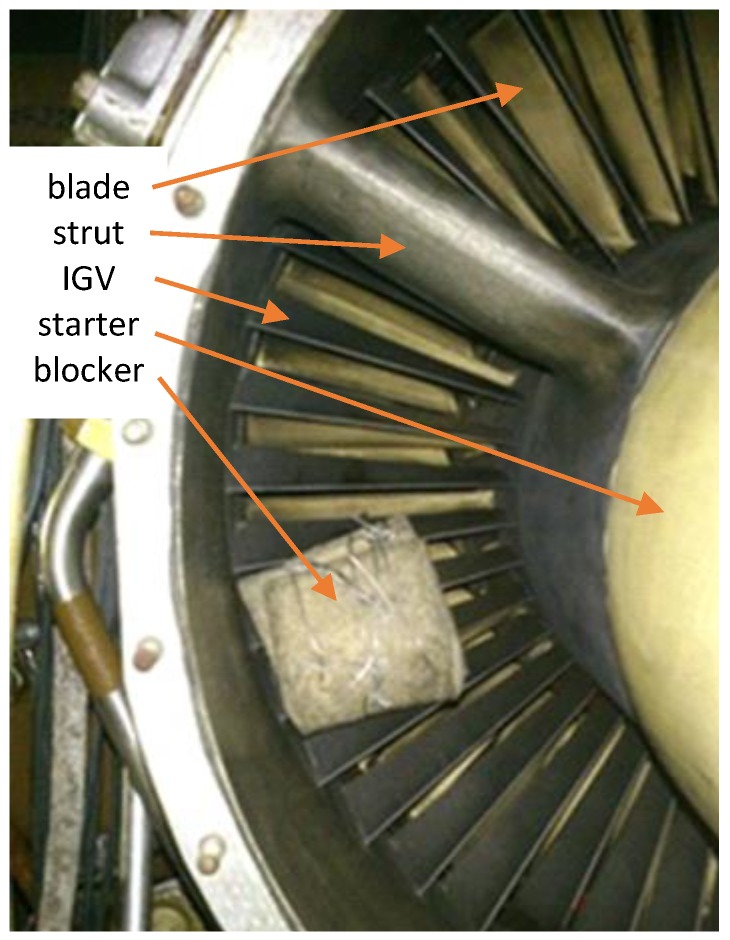
Felt blocker fixed in the inlet during the second test.

The purpose of fitting a felt blocker to the inlet (Figure 2) was not to force any particular order, but to excite as many responses as possible. It significantly increased blade vibration [24] and simulated icing, a bird, or another foreign object ingested and stuck into the inlet guide vanes. Its role was to excite a wide array of resonances, which are normally difficult to measure. Previous research showed [25,26,27] that operation with a foreign object in the inlet results in M1EO2 resonant vibrations exceeding critical stresses and can lead to their fatigue cracking even after several minutes.

**Figure 3:**
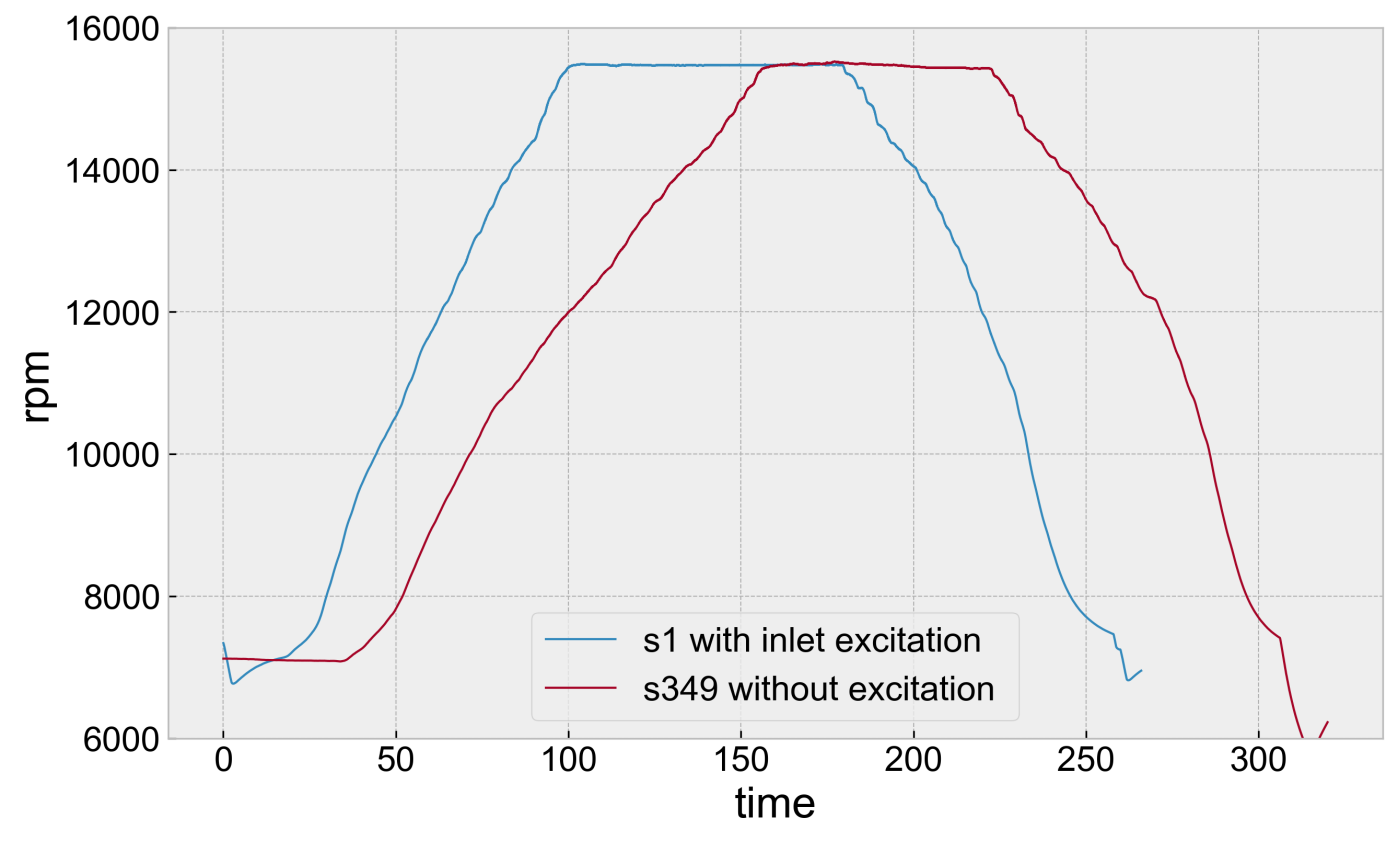
Speed profiles.

Two datasets (Figure 3) are analysed below:

1. Without inlet excitation (S001)
2. With the inlet blocker fitted (S349)

### 2.1. Measurement Setup

**Figure 4:**
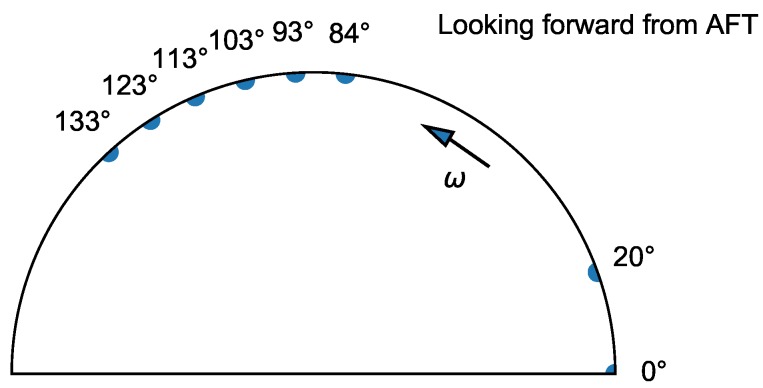
Positions of blade tip-timing (BTT) probes.

The acquired data included signals from eight variable reluctance probes installed in the first stage of the compressor and the once-per-revolution sensor, organised in the form of ’shots’ comprising slow manoeuvres of increasing and decreasing rotational speed. The inductive probes, developed by ITWL, were placed at the following circumferential positions (Figure 4): 0.0, 19.7, 83.9, 93.4, 103.1, 113.1, 122.7, and 132.7 degrees in the slots machined under a previous project. For that reason, sensor spacing could not be optimised.

The data acquisition system was based on National Instruments PXI-1065 computer running a LabView application and two PXIe-6358 modules, which amplified and sampled sensor signals with the rate of 500 kHz. Waveforms were processed in real time by software edge detectors with linear interpolation so the resolution of blade arrival measurement (TOA) is not limited by the sampling rate [28]. The methodology of data acquisition and handling is described in more detail in paper [29].

### 2.2. Model

The blade displacement $d_{j}$, measured at an individual blade for a given probe *j* is of the following form:

(1) $$\begin{array}{cc} d_{j}=P_{j}+a_{0} & +a_{1}sinEO\theta_{j}+a_{2}cosEO\theta_{j}\\ & +b_{1}sinfeo\theta_{j}+b_{2}cosfeo\theta_{j} \end{array}$$

where $P_{j}$ is an invariant displacement offset for probe *j* and is typically due to mechanical variation in the blade positions due to manufacturing tolerances and errors in probe positioning. $\theta_{j}$ is the angular position of probe *j*; EO is an integer engine order for synchronous vibration; $a_{0}$ is a non-probe specific, steady (i.e., non-vibrational) blade displacement offset due to aerodynamic loading and axial position [30], $a_{1}$ and $a_{2}$ are constants from which synchronous vibration amplitude and phase can be calculated; feo is a non-integral (fractional) engine order for asynchronous vibration; and $b_{1}$ and $b_{2}$ are constants from which asynchronous vibration amplitude and phase can be calculated.

For synchronous vibration, Equation (1) can be expressed in the following matrix form:

(2) d=Ma

where $d={\left[d_{1}\;..\;d_{M}\right]}^{T}$, $a={\left[a_{0}\;a_{1}\;a_{2}\right]}^{T}$, *M* - number of probes

(3) $$M=\left|\begin{array}{ccc} 1 & sinEO\theta_{1} & cosEO\theta_{1}\\ \; & .. & \\ 1 & sinEO\theta_{M} & cosEO\theta_{M} \end{array}\right|$$

The system was overdetermined because the number of probes *M* was higher then the number of the vibration parameters a, so the method of least squares can be applied to approximate the solution:

(4) $$\hat{a}={\left(M^{T}M\right)}^{-1}M^{T}d.$$

The algorithms for solving the numerical problem are described by Jousselin et al. [7,22] and Russhard and Back [31]. The operation may be performed in a general-purpose package such as Matlab [10] or Python [29].

The obtained vector $\hat{a}$ is substituted to Equation (2) to compare the calculated displacements $\hat{d}$ with the measured ones d. It is more convenient to use correlation between d and $\hat{d}$ to evaluate the goodness of fit instead of the residual $d-\hat{d}$. The correlation coefficient *r* is calculated using the Pearson formula:

(5) $$r=\frac{\sum \left(d-\bar{d}\right)\left(\hat{d}-\bar{\hat{d}}\right)}{\sqrt{\sum {\left(d-\bar{d}\right)}^{2}{\left(\hat{d}-\bar{\hat{d}}\right)}^{2}}}$$

This parameter ranges from 0 to 1 and is called ’coherence’ below.

lEstimated vibration amplitude equals:

(6) $$\hat{A}=\sqrt{{\hat{a}}_{1}^{2}+{\hat{a}}_{2}^{2}}$$

Traditionally, peak-to-peak amplitude is usually produced by tip timing systems for comparison with deflection charts:

(7) $${\hat{A}}_{\mathrm{pk}-\mathrm{pk}}=2\hat{A}=2\sqrt{{\hat{a}}_{1}^{2}+{\hat{a}}_{2}^{2}}$$

### 2.3. Stack Pattern

From Equation (1) it can be seen that for a blade undergoing no vibration, its steady state displacement is equal to $P_{j}$. For an ideal rotor with equally spaced blades, the value for each blade would be identical. In reality, the difference in manufacturing tolerances produces a pattern of displacements, which are the differences from the ideal position. It is unique for each rotor and shown as the stack plot [7]. It is calculated for each probe *j* by averaging blade displacements over many revolutions:

(8) $$P_{ij}=\frac{1}{H}\sum_{h=1}^{H}d_{hij}$$

where *h* is the revolution number, *i*—the blade number, *H*—the number of revolutions.

Changes in the stack pattern occur if (a) the blades undergo vibration [32] or (b) if the rotor is permanently damaged for some reasons. The stack plot is used to verify that the collected data sets are aligned correctly and is often applied as the first level of data validation. The analysis of misaligned data is a common fault in BTT data analysis.

### 2.4. Software

Blade deflection signals include undesirable components, such as noise, a static offset, a linear trend, and also some asynchronous vibration, which is not of interest. Conventional processing of BTT data relies upon preliminary operations such as alignment to ensure all data comes from the same rotation number, zeroing to isolate $P_{j}$ and filtering to isolate the integral response from the non-integral response [33].

In practice, the number of resonances, blades, and probes to analyse is sufficiently large that pre-processing and resonance fitting must be automated. The automation of analysis was achieved by Rolls Royce, which developed their Batch Processor software [34], which was released several years ago. The solver separates the integral and non-integral components of the blade displacement and uses a form of least squares fitting to generate the results for integral and non-integral responses. Where integral and non-integral displacements precede and follow on an integral response, or occur simultaneously, then that software fails.

In this work, a new solver called EMTD Multitool [35] was used. It has the ability to operate conventionally or by using alternative methods of pre-processing the data, which can be manipulated into the form:

(9) $$d_{j}=a_{0}+a_{1}sinEO\theta_{j}+a_{2}cosEO\theta_{j}$$

where the value of EO is real number rather than an integer.

### 2.5. Uncertainty Measurement

BTT uncertainties can be divided into three categories: those inherent in the physical act of taking the measurement, the pre-processing of the acquired data including the conversion to deflection and frequency, and finally, the challenge of converting the obtained deflections into blade stress [36]. The current capability of BTT for use in a compressor stress survey can, if all of the above uncertainties are addressed and controlled, produce end-to-end stress measurement with uncertainties of ±2.5% or better. More importantly, a figure of uncertainty can now be calculated for every BTT measurement.

Typically, BTT responses are characterised by the quality of the least squares fitting. However, measurement uncertainty is often not known and the decision on whether the results are ‘good’ is subject to opinion. The calculation of the uncertainty value removes this doubt and provides upper and lower values to any BTT measurement.

The MultiTool solver calculates the measurement uncertainty for any point on the tracked order response. To do this, the least squares model is extended to contain a term that ‘captures’ any data that do not correlate with the engine order term. The uncertainty term includes the average and standard deviation of the non-related terms. From this, the measurement uncertainty is calculated over a pre-determined number of revolutions. The form of unc is not disclosed in this paper for commercial reasons.

(10) $$d_{j}=a_{0}+unc+a_{1}sinEO\theta_{j}+a_{2}cosEO\theta_{j}$$

### 2.6. Data Analysis

**Figure 5:**
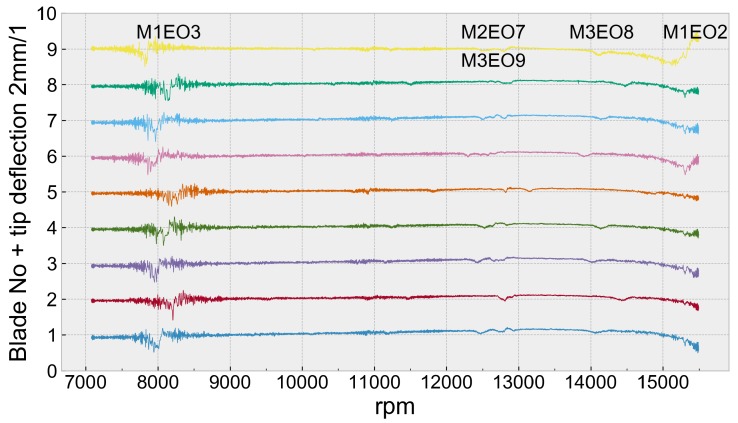
Tip deflection measured by sensor one as a function of rotational speed after low pass filtering (S349).

Integral responses were initially identified in the graph of the filtered blade displacement signal, coming from a selected sensor (Figure 5). Circumferential least squares fitting was applied to the observed vibration events. The engine orders to be fitted in the selected speed ranges were defined not only thanks to past experience, but also FEM modelling [37,38], prediction of likely vibrational modes and spectral analysis.

**Figure 6:**
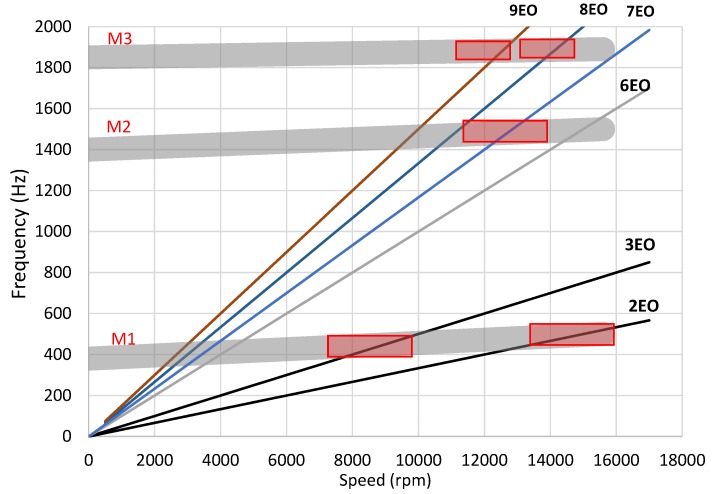
Campbell diagram: lines of synchronous excitation (spokes), predicted zones of vibratory modes M1–M3 and expected resonances.

The approximate values of vibration frequency for selected modes and speeds were taken from the results of numerical modal analysis [39]. On the basis of this data, a Campbell diagram was plotted, which was necessary to set up the solver (Figure 6). The observed resonances M1EO2, M1EO3, M2EO7 and M2EO8 were fitted in the so-called ’synchronous analysis’ with the integral engine order. The selection of resonances is limited by the available speed range (7200–15,500 rpm) and the set of engine orders which is generated by the excitation force.

It can be seen that the M1EO2 and M2EO6 resonances (15,500 rpm) and also M2EO7 and M3EO9 (12,500 rpm) were close together, so they may have required a double analysis, i.e., a model with two degrees of freedom. In the case of two simultaneous responses, a further term was added to the blade tmodel:

(11) $$\begin{array}{cc} d_{j}=a_{0} & +a_{1}sinEO_{1}\theta_{j}+a_{2}cosEO_{1}\theta_{j}\\ & +a_{3}sinEO_{2}\theta_{j}+a_{4}cosEO_{2}\theta_{j} \end{array}$$

It is worth noting that the resonances M1EO3 and M2EO8 coincided with asynchronous vibrations occurring below 12,000 rpm. It could affect the estimation using the conventional solvers as they allow for fitting the selection with fixed orders only.

To analyse coinciding integral and non-integral vibrations, an alternative solver was employed which tracked the modal frequency in the band defined by the Campbell diagram, both in a steady state and transient mode. Fourier spectrum delivered the initial EO value for the measured displacements which was used for least square fitting regardless the type of the response. The engine order was then refined and the process repeated until satisfactory coherence was achieved.

The solver produced the sequence of amplitude and phase values estimated for subsequent revolutions, stored in spreadsheet files. The further sections deal with the processing of fitting results and interpreting them.

## 3. Results

### 3.1. EO2 Forced Response

The SO-3 turbojet had a low order resonance in take-off range which is an obvious design error. It was observed that the inlet blocker doubled the amplitude of M1EO2 and other responses occurring above 12,000 rpm. The deflection curve at the take-off speed 15,500 rpm showed the blade leaning as it went towards resonance (Figure 5).

The stack pattern of the BTT data sets was used as a first level validation of the data. If carefully managed, it could be used as real time indicator of integral vibration reinforcing the dangers of looking at raw displacement curves to the untrained operator. In the case there are discrepancies, the analysis software should refuse to load incorrect data.

**Figure 7:**
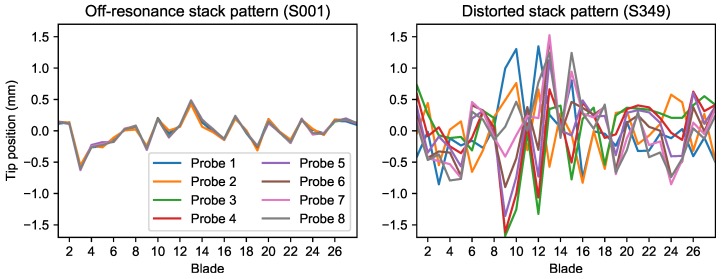
Stack plots.

For s349 data-set, the extreme forced vibration reaching 3 mm pp distorted the stack plot (Figure 7). The mean values calculated for the stack deviated as a result of high integral vibration and consequently, the alignment checks on the stack pattern failed. Batch processor reported the stack pattern’s error and denied loading and analysing the shot S349. There was no standard approach for cases when automated zeroing failed. Some software packages, including MultiTool, allow the data to be corrected for errors before allowing analysis. Both programs are unable to process this resonance properly without careful configuration requiring an understanding of BTT methods.

**Figure 8:**
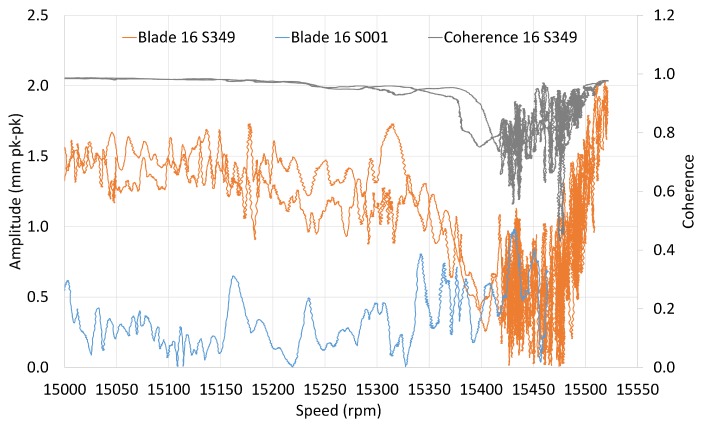
M1EO2 tracked order for a single blade.

The fitting results of the amplitude and phase of individual blades are available in spreadsheet files for further processing. Figure 8 shows the tracked order overlay for a single blade with and without the blocker. The same maximum speed was not reached for both setups, but a forced response was observed on the blade prior to approaching its resonant speed, as well as increased instability caused by the blocker. However, the resonance was not achieved in both cases due to speed limitations.

**Figure 9:**
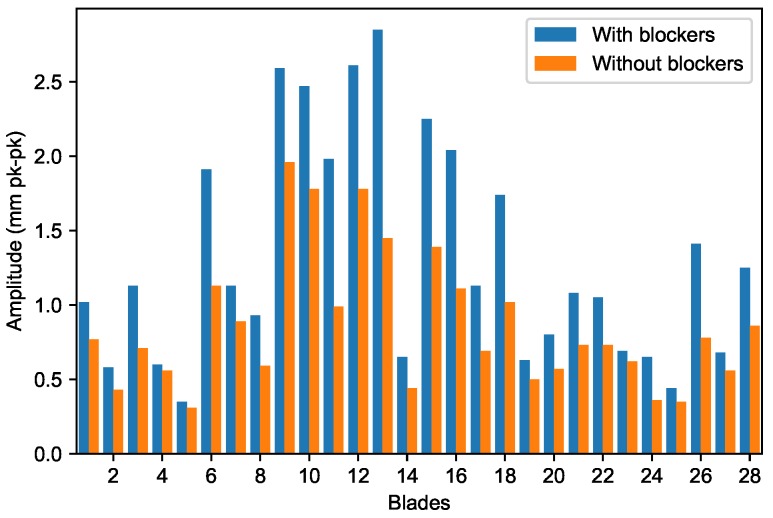
M1EO2 peak amplitude response for all blades.

The summary plot (Figure 9) shows that several high-responding blades were present, for which the M1EO2 resonance was very close. Ease in exciting them to excessive vibration posed a threat to the engine’s durability. However, the mistuning pattern was very similar for the both tests (Figure 9) which confirmed that the blades were still healthy. The amplitude mistuning pattern can be applied to monitor blade health, similarly to the frequency one [40].

As discussed by Heath and Imregun [41], fitting results indicate that the real tip deflection is ten percent greater than the peak amplitude of zigzag read from the single sensor chart (Figure 5). This kind of a coarse measurement, known as Zablotskiy method [42], should, therefore, be used only as a first approximation, while reliable synchronous vibration measurement requires a multi-sensor system. This is extremely important as viewing displacement plots for blades provides very little information but is often considered by engineers as an essential tool e.g., [3,43,44].

### 3.2. Frequency Estimation

In theory, vibration phase changes the sign when crossing the resonance but a 180-degree phase shift is observed in rare cases and cannot be used reliably to confirm the location of resonances. In a real test environment, examining each phase response visually is impractical even for low-order responses, which need many revolutions to sweep. If the phase shift was used as the only indicator defining the resonance, then less than 1% of existing resonances would be found in real engine datasets.

Mostly, it is assumed that the blade resonant frequency occurs at the point where amplitude reaches a maximum value. The quality factor of metal blades is high (e.g., 200 or more) so the resonance peak is a good approximation of the resonant frequency. The frequency measurements presented in the next section are obtained in this way. Unfortunately, the M1EO2 resonance was located in the vicinity of the upper-speed limit. As a result, no phase transition by zero was observed for the majority of blades, which means that the resonant frequency was not achieved, so it could not be reliably determined. Therefore, the values presented in Figure 9 correspond to the maximum amplitude reached by the solver.

In the case when full resonance is not achieved, erroneous results can be produced, although this is the same as with strain gauges. For M1EO2, checking the phase shift helps to avoid presenting false frequencies. In other cases, it is usually not necessary and could remove potentially good data, if the threshold is increased over 105 degrees.

Here, there was no simple method of measuring the resonant frequency of the blades whose M1EO2 resonance was located beyond the maximum speed. The solution is to increase the maximum engine speed but it reduces engine durability and is not recommended for safety reasons.

### 3.3. Higher Order Resonances

**Figure 10:**
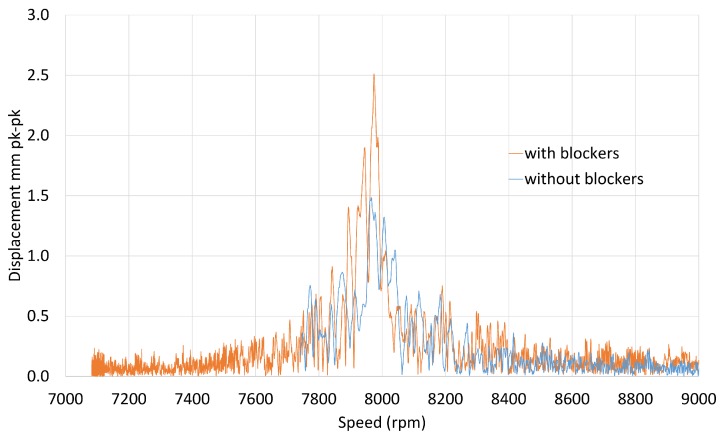
Single blade response—M1EO3.

**Figure 11:**
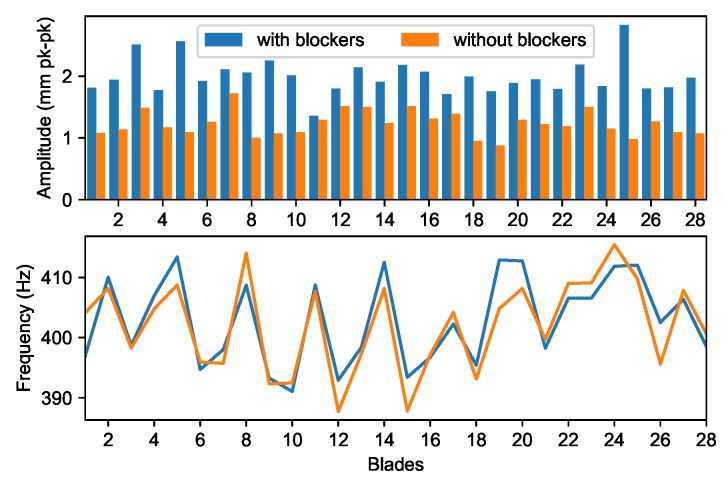
M1EO3 peak amplitudes and frequencies for all blades.

The increase in amplitude caused by the blocker was also observed for the M1EO3 crossing at 8000 rpm (Figure 10). The tracked order blade response showed the increase and decay of the amplitude. The resonance was traversed during the engine manoeuvre, so the blade response frequencies can be compared (Figure 11). The uncertainty of the presented resonant amplitudes was limited to 5% and is around 1% for the majority of blades. Therefore, error bars are not displayed in summary plots to avoid blurring them.

**Figure 12:**
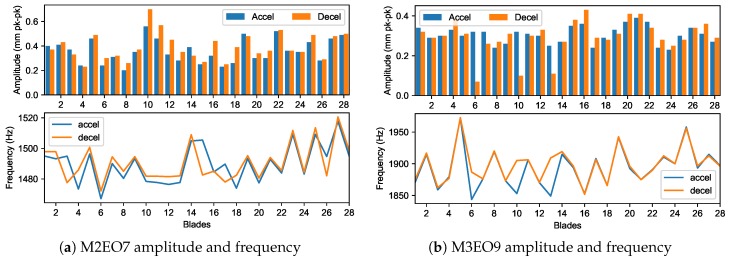
Double analysis.

The blade amplitudes of the other resonances predicted by the Campbell diagram can be also extracted. It was apparent that integral M2 and M3 vibrations responded well only when the blocker was installed (shot s349). Figure 12a shows M2EO7 observed at 12,800 rpm which may be coincident with M3EO9, thus it was calculated as a double response. Similarly, the results from the double analysis for M3EO9 are shown in Figure 12b. This method involved factorisation of a larger matrix and thus took significantly more time than the single analysis.

**Figure 13:**
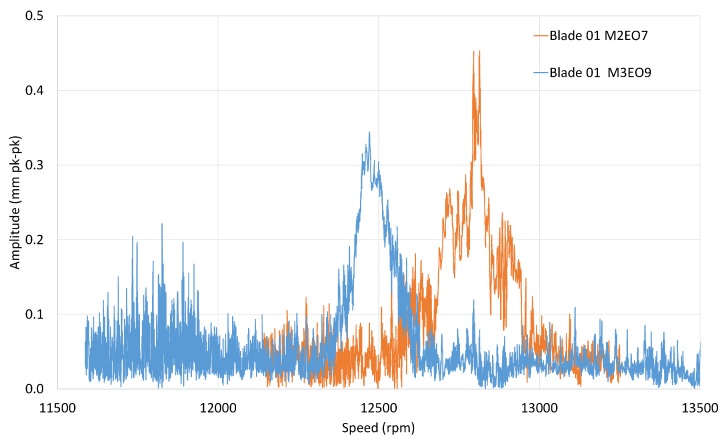
M2 and M3 overlay shows no coupling.

Observing the responses and overlaying them on a common speed time base (Figure 13) indicates that the two modes were not really coupled and could have been analysed as single responses.

**Figure 14:**
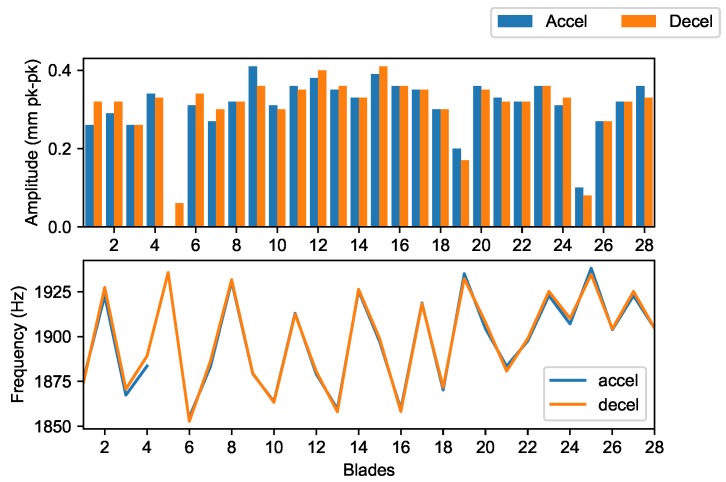
M3EO8 amplitude and frequency.

**Figure 15:**
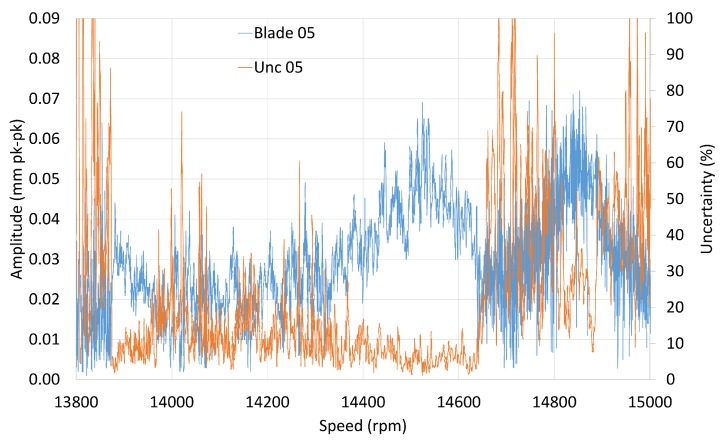
M3EO8 amplitude uncertainty.

At about 14,000 rpm, another M3 resonance was observed (M3EO8), which was analysed as a single response and gave similar results as M3EO9 (Figure 14). A weak response from blade 5 for acceleration was masked by the use of a coherence and uncertainty filter. Measurement uncertainty can be used to assess the overall results because it is calculated for any point on the tracked order response (Figure 15).

Lowering the thresholds for the filters would then reveal the amplitude for blade 5. However, its uncertainties would be outside of the limits set by the user. In reality, it indicates that a clean response from this blade cannot be obtained and for this particular manoeuvre can be ignored.

The mistuning patterns presented in this section, both amplitude and frequency, are intended to be used for monitoring blade health. BTT methods provide data from many blades unlike the strain gauges, so it is acceptable to ignore ‘noisy responses’. However, in the case where the blade is always unable to produce the expected response, a more detail post–test inspection of the blade would be pertinent.

Responses of higher modes than M3 and orders greater than nine, despite being predicted by FEM modelling, did not respond sufficiently for most of the blades to meet required fit quality and uncertainty levels. A more selective excitation method such as oil jets or electromagnets is necessary to investigate them. Probe positioning has to be redesigned to cover high orders and complex mode shapes. Limited spatial resolution of the inductive sensor could be a barrier [45]. Another problem with higher orders is that they last only for a few revolutions, so an accurate FEM model and more precise speed control are needed to find them.

### 3.4. Asynchronous Vibration

**Figure 16:**
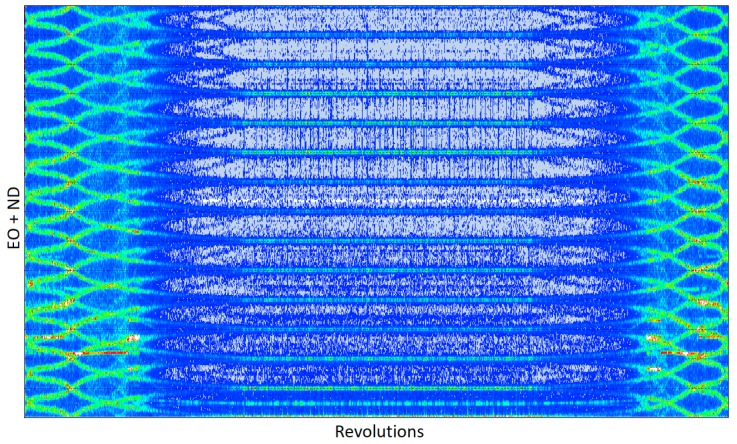
Travelling wave plot.

The compressor is not equipped with guided vanes, so there are rotating stall and other intensive asynchronous vibrations observed below 12,000 rpm that disappear at higher speeds. This is shown in the all-blade spectrum plotted for the s1 dataset (Figure 16).

When the speed changes (the left and right part of Figure 16), vibrations of individual blades are visible in the spectrum in the form of a crossing of the engine order axis of the diagram. Asynchronous vibration is presented as a coherent trace over the blue background denoting the noise floor. The average assembly amplitude and nodal diameter of travelling wave responses can be found by observing the vibration from two different angular positions [46,47,48]:

(12) $$ND=\frac{\phi_{2}-\phi_{1}}{\theta_{2}-\theta_{1}}$$

**Figure 17:**
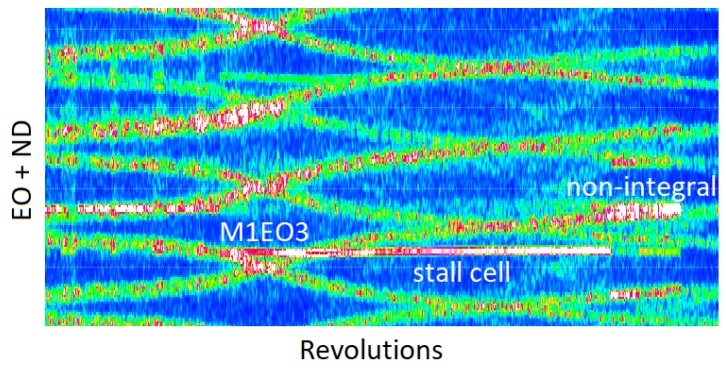
Zoomed travelling wave plot.

$\phi_{j}$ is the phase of the spectral line observed by probe *j*. Figure 17 shows a zoomed region showing a rotating stall response, which arises during ramp-up at the idle speed and exists up to 11,500 rpm. This is M1 vibration with the nodal diameter -4 ND and 1.8 EO.

**Figure 18:**
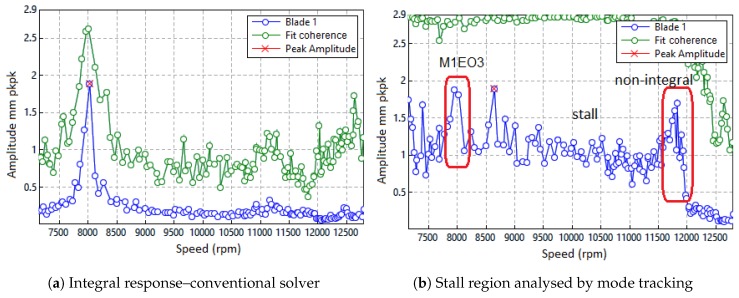
Conventional solver vs. mode tracking.

The data produced so far has used the conventional pre-processing methods of separating both the integral and non-integral terms through a filtering and zeroing process and then performing least squares fitting that is seeded with the estimated response engine orders. If the conventional integral solver is applied to the region shown in Figure 17, only the peak response at the resonance can be extracted. The software is not able to change the order on the fly so the fitting fails for speeds higher than 8500 rpm (Figure 18a).

**Figure 19:**
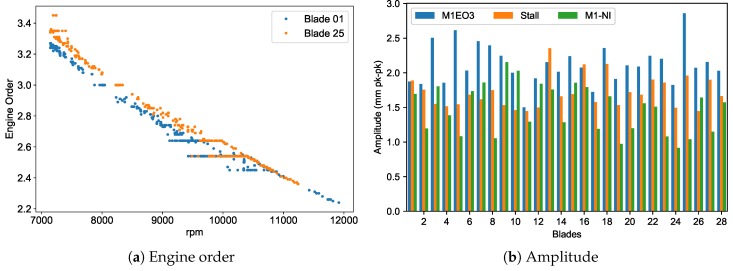
Successive M1 responses analysed using modal tracking solver.

By using alternate methods to process and fit the data, the simpler model (Equation (9)) can be applied irrespective of the type of vibration. It delivers the mode tracked response similar to that which is produced from strain gauges. When applied to the region of interest, the modal solver reveals the peak amplitude which occurred in the stall for some blades and that the stall collapsed at 12,000 rpm (Figure 18b). For the three successive responses observed in the region i.e., the M1EO3 resonance, stall cell and natural M1 non-integral response, the peak amplitudes of individual blades are obtained (Figure 19b). Both methods i.e., the traditional fixed-order LSF and proposed modal solver, give similar results for homogeneous responses. The differences can be attributed to the pre-processing filtering and zeroing techniques used.

## 4. Discussion

Vibration analysis is necessary for designing compressors and turbines in a way that reduces the risk of high-cycle fatigue of the material, which under certain conditions can lead to cracking and breaking of the blades. Knowing the real dynamic properties of the blades is crucial both for designers and users of gas turbines. In this example, it is shown that complex modes of vibration such as simultaneous resonances and travelling wave responses can occur in any compressor, even in a legacy turbojet of a basic structure.

The problem of the excessive vibration of SO-3 fan blades in the take-off range is well understood and M1EO2 responses are visible on the tip-deflection traces without any additional measures. Even for such a basic response, reading deflection charts is imprecise and largely affected by the human factor. Only model-based vibration analysis can be used when developing and certifying components.

The BTT method has its limitations and can only partially replace the strain gauges [9]. For example, it is difficult to measure higher modes of vibrations, whose deflections in the direction of rotation are of a few microns. If they are accompanied by significant stresses, they can be observed with strain gauges, but there are also problems with transmitting the signals of several kilohertz frequencies by the telemetry system and the effect of the strain gauge on high-frequency responses.

Observing coinciding responses with the non-intrusive method requires a good understanding of dynamics based on FEM models and proper preparation of the analysis. Higher-order modes can be measured providing that sensors with a suitable spatial resolution are used, the circumferential and axial distribution of the sensors is optimised and the method of exciting vibrations is effective. Many responses which are numerically predicted are extremely difficult to be excited in a test cell. They can be observed only in compressor rigs or vacuum spin facilities equipped with sophisticated excitation systems.

Using the maximum value of the resonance curve gives a good approximation of the resonant frequency. In a few cases, it can be determined based on the speed, at which the vibration phase passes through zero. The obtained frequency charts show that the blades are ordered by their natural frequency during assembly to ensure the differences in the neighbourhood of ±2%. Despite the high mistuning, a travelling wave response was observed with an average assembly amplitude of around 1.5 mm pp, which was part of the rotating stall occurring due to the lack of variable guided vanes.

## 5. Conclusions

The recent version of least squares analysis, handling non-integral engine orders, is an effective method of estimating synchronous and non-synchronous vibrations that can cope with real signals including noise. The presented solver can track the modal frequency and adjust the engine order on the fly. The obtained results are constantly evaluated by monitoring coherence and uncertainty. This procedure let us analyse the three close M1 responses (integral, stall and non-integral) in one go and saved a lot of the tedious work configuring the conventional solver, in which the integer and non-integer responses are treated separately. By using the same method for both types of vibration, it was possible to examine the interaction between the rotating stall and the M1EO3 resonance. This generalised approach to blade vibration analysis and uncertainty estimation is an important step towards replacing strain gauges in engine certification.

## Abbreviations

The following symbols and abbreviations are used in this manuscript:

$\phi_{j}$	phase observed by probe *j*
$\theta_{j}$	angular position of probe *j*
*A*	blade vibration amplitude
$d_{j}$	blade displacement measured by probe *j*
feo	fractional engine order
*H*	number of revolutions
*h*	revolution number
*i*	blade number
*j*	probe number
*N*	number of blades
*M*	number of probes
$P_{j}$	invariant offset for probe *j*
pk-pk	peak-to-peak amplitude
*r*	Pearson correlation coefficient (coherence)
rpm	revolutions per minute
unc	uncertainty
BHM	Blade Health Monitoring
BTT	Blade Tip-Timing
EO	Engine Order
FEM	Finite Element Method
FFT	Fast Fourier Transform
IGV	Inlet Guide Vanes
ITWL	Air Force Institute of Technology in Warsaw
LSF	Least Squares Fitting
M1	1^st^ vibration mode
MDPI	Multidisciplinary Digital Publishing Institute
ND	nodal diameter
NI	non-integral vibrations
NSMS	Non-contact Stress Measurement System
